# Experiences of living with, managing, and preventing reoccurrence of Diabetic Foot Ulcers: Restoring context and complexity to health and illness research

**DOI:** 10.1177/13634593251387548

**Published:** 2025-11-08

**Authors:** Ruth I. Hart, David Rankin, Fran Game, Kavita Vedhara, Julia Lawton

**Affiliations:** 1 Usher Institute, Edinburgh, UK; 2 University Hospitals of Derby and Burton NHS Foundation Trust, UK; 3 Cardiff University, UK; 4 Keele University, Staffordshire, UK

**Keywords:** qualitative research, diabetes, diabetic foot ulcer, lived experiences, context

## Abstract

Diabetes is a major global health concern, with diabetic foot ulcers (DFUs) presenting a common complication. Interest is growing in people’s lived experiences of DFUs, including their management and prevention. Typically, research has highlighted the disruptive and extended impact of DFUs, and has focused on the role of intrinsic, individual factors (e.g. knowledge, physical capabilities and personal choices) in their development and management. The influence of extrinsic, contextual factors, has received comparatively limited attention. To address this potentially important gap in the literature we set out to explore people’s experiences of DFUs, using a distinctive, contextually-sensitive, analytical lens. Our analysis led us to identify three over-arching themes: a *spectrum of embodied experiences of DFUs*; *intersection with wider experiences of ill-health*; and, *framing of (DFU) experiences by broader life circumstances*. Within these themes, considerable diversity was evident. Broader life circumstances, in particular, shaped experiences of living with, managing, and preventing reoccurrence of DFUs in markedly different, and previously unacknowledged, ways. We conclude that experiences of DFUs are far more nuanced and contextually-mediated than previously reported, and identify important practical implications for the provision of sensitive and effective support and clinical care. Moreover, we suggest that identifying these sorts of complexities in illness experiences may require larger, and more varied samples and data-sets than have become the norm in qualitative health research, as well as more expansive disciplinary lenses.

## Introduction

Diabetes is a chronic metabolic disorder that alters how the body reacts to or produces insulin. It affects substantial and growing numbers of people globally, including more than 4,500,000 in the United Kingdom (UK). Around 90% of those affected have type 2 diabetes (T2D); a further 8% have type 1 (T1D; Whicher et al., 2020). Despite important therapeutic advances, diabetes remains challenging to manage. Individuals with T2D are encouraged to make dietary changes, increase physical activity, manage their weight and, if their blood glucose levels remain high, take glucose-lowering medications. For those with T1D, insulin is the mainstay of treatment; individuals need to adjust doses in light of blood glucose levels and other factors, including food intake, exercise, and illness. Many people struggle to keep their glucose levels within clinically-recommended target ranges. This places them at increased risk of micro- and macro-vascular complications, including heart disease, stroke, renal failure, blindness and diabetic foot disease (Whicher et al., 2020).

Diabetic foot ulcers (DFUs) are a common and potentially serious complication which affect an estimated 19%–34% of people with diabetes (Armstrong et al., 2017). Whilst DFUs vary in severity and acuity, rates of morbidity and mortality are high. Infection is common and leads, relatively frequently, to lower extremity amputations (Rodrigues et al., 2022). Over 40% of people presenting with DFUs die within 5 years (Walsh et al., 2016). In short, DFUs generate substantial personal and healthcare costs (Kerr et al., 2019). People with diabetes are therefore advised to adopt a suite of risk-management strategies. These include: wearing well-fitting and protective footwear; conducting daily foot-checks; and, seeking professional help promptly on identifying abnormalities (Diabetes UK, 2024).

Interest is growing in the experiences and perspectives of people who have had DFUs or are at risk of these, with research highlighting the distressing and disruptive nature of ulceration (see, e.g. Barg et al., 2017; Beattie et al., 2014; Costa et al., 2024; Costa and Camargo-Plazas, 2023; Marchand et al., 2012; Searle et al., 2008). Indeed, having synthesised 42 papers published up to 2016, Coffey et al. (2019:208) concluded that the physical, social, and psychological impacts of DFUs appeared to be ‘significant and enduring’. Subsequent publications have largely reinforced this characterisation (e.g. Costa et al., 2024; Costa and Camargo-Plazas, 2023; Crocker et al., 2021; Meriç et al., 2019). However, some recent work suggests that experiences may be more varied. Specifically, Aagaard et al. (2024: 432) reported how their interviewees, in contrast, depicted their DFU experiences as merely a temporary setback, or ‘bump in the road’. Whilst that study was small-scale and single site, this alternative characterisation suggests that experiences of DFUs may be more diverse than previously reported.

It is notable that most work to date has focused on the role of intrinsic, individual factors in foot-care and consequent foot-health. Coffey et al. (2019) highlighted how synthesised work suggested that *knowledge* of DFUs and protective foot-care was often limited or incorrect, and encouraged unhelpful or risky practices. Their synthesis also drew attention to the ways in which people’s footwear *preferences, desire* to sustain ‘normal’ lives, and physical *capabilities*, had detrimental impacts on their foot-care decisions and behaviours. Subsequent work has largely reinforced these kinds of observations, by highlighting how a lack of knowledge, understanding and/or awareness (of disease processes, risks and/or protective factors) can negatively affect foot-care behaviours, foot-health experiences and outcomes (Barg et al., 2017; Meriç et al., 2019; Simonsen et al., 2024; Van Netten et al., 2019).

This focus on individual intrinsic factors likely reflects a legitimate concern, in some disciplines, with factors judged as potentially modifiable. However, the role of conceivably important external factors, including access to specialist services and resources, has been far less frequently considered (Costa et al., 2020; Littman et al., 2021 being notable exceptions here). Studies of DFU experiences and foot-care behaviours which are rooted in the wider conditions of people’s lives remain hard to locate. Yet sociologically-informed research on experiences of health and illness suggests that such a contextually-sensitive approach may be both important and illuminative. Indeed, in her seminal monograph, Cornwell (1984: 123) argued that peoples’ accounts of health/illness could ‘only be properly understood in the context of lives as a whole’, including their social and material realities (i.e. tangible, physical conditions). Whilst the extended ethnographic work Cornwell undertook is beyond the ambitions and/or funding of most contemporary health researchers, the idea that context matters has gained increasing traction over time. Indeed, it now finds support in a range of disciplines, including realist(ic) evaluation (Pawson and Tilley, 1997), medical sociology (Lawton, 2003), improvement science (Bate, 2014), and applied health services research (Rogers et al., 2020).

Hence, to address marked and potentially important gaps in the literature, we explored people’s lived experiences with a distinctive analytical lens. This was sensitive to extrinsic factors, and grounded firmly in the contexts of people’s wider health and lives. In so doing, we sought to advance understandings of experiences of living with, managing and preventing DFUs, and to offer recommendations to improve the support and clinical care provided to affected individuals.

## Methods

We outline our methods (and report our findings) broadly in accordance with the consolidated criteria for reporting qualitative studies (COREQ; Tong et al., 2007). Supplemental File 1 signposts readers to information relevant to each of the 32 COREQ criteria.

### Design and context

We undertook a large qualitative study in conjunction with two linked, multi-centre randomised controlled trials (see https://www.isrctn.com/ISRCTN15460422 and https://www.isrctn.com/ISRCTN15570706). That qualitative study included interviews with trial participants – all of whom were individuals with recently healed DFUs. The two trials (a pilot and main trial) tested iterations of a complex psychosocial intervention, ‘REDUCE’, designed to reduce DFU reoccurrence and escalation. Whilst our qualitative research was longitudinal, we focus here on data from ‘baseline’ interviews, conducted after randomisation, but before participants received the intervention. Philosophically, the qualitative work was informed by a pragmatic, ‘common-sense’ critical realism (discussed further, below); more specifically, the ontological position that an external (physical and social) reality exists, independent of our (and our interviewees’) knowledge of it, combined with the (essentially constructivist) epistemological position that our interviewees’ (and our own) understandings of and perspectives on experience are socially-mediated, situational, and incomplete (Maxwell, 2012). Methodologically, our work, like much applied qualitative research, was loosely informed by principles and procedures of grounded theory (Strauss and Corbin, 1990). For instance, the influence of the grounded theory tradition can be seen in: the inductive (inclusive and substantially data-led) nature of our work; our iterative approach to data collection and analysis; our highly systematic approach to coding, exemplified by our use of the technique of constant comparison; and, finally, the ways in which the act of writing supported the development and refinement of our analytical thinking.

### Participants and recruitment

Potential participants in the trials – and by extension the qualitative research – were identified, screened and recruited by diabetic foot-care teams at 27 NHS Trusts around England. To be eligible, individuals needed to be: ⩾18 years of age and have: a diagnosis of diabetes; two lower limbs (no major amputations); a recently healed DFU; and, sufficient cognitive capacity and command of English to engage with the trialled intervention. On enrolling in the relevant trial, participants provided written informed consent to take part in the nested qualitative research (consent was also confirmed verbally before any interviews began). A sub-set of trial participants who had been randomised to the intervention arm were subsequently selected for participation in the qualitative research. Where possible, we sampled purposively, using criteria which, based on and our own and others’ research, we anticipated might affect people’s experiences and/or perspectives. This included sampling on the basis of: recruitment/clinical site (Lawton et al., 2005); age (Lawton, 2002); gender (Emslie et al., 2001; Hjelm et al., 2002); and, previous DFU (one only, or more). Ideally, we would also have sampled by: type and duration of diabetes (Lawton et al., 2008a); socio-economic status (Cornwell, 1984); and, ethnicity (Lawton et al., 2006a, 2008b). However, we lacked access to reliable, relevant information prior to undertaking the interviews. Instead, we monitored those characteristics as we collected data, continuing recruitment until our sample was sufficiently diverse to support a contextually-sensitive analysis.

### Data collection

As noted above, our work was influenced by critical realism, an approach encouraging close attention to ‘real bodies . . . and real worlds’, as well as to the meanings attached to illness and disability (Williams, 1999: 797). This approach informed initial interview topic guide development, along with other relevant literatures (outlined above), our own prior experience of evaluating behaviour change interventions and input from clinical and behavioural science colleagues. We also consulted members of the REDUCE Patient and Public Involvement and Engagement group at this stage (cognisant of the research team’s lack of direct, personal experience of living with, managing, and preventing reoccurrence of DFUs). Importantly, we took an iterative approach to data collection and analysis, reviewing and revising our topic guide (content and structure) as work progressed, both on transitioning from the pilot to main trial and throughout the course of the main trial. Whilst Box 1 presents the core topics addressed in interviews, the guide both evolved over time and was employed flexibly and responsively. A conversational style was adopted and interviewees were enabled and encouraged to raise topics and share information they deemed important. Interviews with sampled participants were negotiated and/or arranged by the REDUCE programme team alongside scheduling intervention sessions. Two experienced qualitative researchers (RIH and DR – one female and one male, both non-clinical and independent of the trial team) conducted the interviews, by telephone or video-call as interviewees preferred. Interviews lasted 45–90 minutes. All were digitally recorded and transcribed verbatim. Baseline interviews with pilot trial participants took place between June and September 2021, and those with main trial participants between May 2023 and January 2024.

**Box 1.** Indicative topic guide.

**Interviewees’ backgrounds and daily life** • Demographic characteristics • Domestic and work circumstances/arrangements • Routines and responsibilities day-to-day and week-to-week • Key relationships and social connections • Interests e.g. leisure and social activities **Health in general** • Physical health (issues/conditions other than diabetes and their impact) • Mental health (associated with and/or distinct from physical health issues) • Diabetes (type, duration of diagnosis, management, challenges encountered, other complications, in-/formal support received, impact on life) **Foot-health and care** • Recent DFU experience (when/how it developed; how they became aware it was a problem; what they did and/or others did; life with, and the impact of, an active DFU; time to/conditions of healing) • Other/historic experiences of DFUs • Education and knowledge about foot-health and care (including any changes since having a DFU and/or over time) • Foot-health and care practices e.g. foot-checking, foot-wear, weight-bearing activity (including any changes since having a DFU and/or over time) • Challenges to optimal foot-care and health, if any • Support for optimal foot-care and health, if any (formal and informal) • Expectations of/for future foot-health and care **Making changes to improve health** • Anything they do currently to maintain health (in general) e.g. diet, activity • Experiences of changing behaviour for health reasons (e.g. giving up smoking) • What made/makes adopting ‘healthy’ behaviours easy/ier and hard/er • Sense of potential to improve one’s health (control over health outcomes) • Expectations of/for health going forward (including anticipated impact on life of diabetes) **Experience of difficult feelings** • Anything other than health that affects enjoyment of life (e.g. financial concerns, relationship difficulties, care responsibilities, insecure or stressful work) • Approach to dealing with challenges, difficult situations, and/or setbacks • Impact/influence of mood on experience and behaviours • Strategies for dealing with low mood and/or difficult feelings **Expectations of REDUCE** • Reasons for taking part in the trial (any expectation of benefit?) • Any questions or concerns • Feelings about being randomised to the intervention arm • Anticipated challenges or barriers to completing the intervention **Anything else the interviewee judges salient**

### Data analysis

Critical realism recognises context as playing a pivotal role in explanation(s), hence we took an inclusive and data-led approach to thematic analysis (Braun and Clarke, 2006). Data were analysed by three experienced qualitative researchers (all non-clinical and independent of the trial team): RIH, DR and JL. Through close and repeated reading of transcripts, these researchers individually identified areas of interest and provisional, preliminary themes. They then met to share interpretations; differences were minimal and easily resolved through discussion. Once consensus had been reached, RIH systematically coded and collated data relevant to three agreed overarching themes. Using the technique of ‘constant comparison’ (Strauss and Corbin, 1990), the collated data were then further scrutinised, compared and re-coded, to refine those themes and identify analytical sub-themes. These were documented and illustrated in analytical reports, which were discussed within the team and provided the foundation for the analysis we present below. Clinical co-authors ‘sense-checked’ data interpretations and contributed to the recommendations presented in the Discussion.

## Findings

We interviewed a diverse range of participants (*n* = 35) from 12 trial sites; see Table 1 for more information on our sample. We report below how these interviewees (all given pseudonyms) described their lived experiences of DFUs, including factors affecting management and prevention. Through analysis, we identified three over-arching themes. Variation within these themes underpinned marked differences in overall experiences and perspectives. The themes were: (1) a spectrum of embodied experiences of DFUs; (2) intersection with wider experiences of ill-health; and, (3) framing of (DFU) experiences by broader life circumstances.

**Table 1.** Characteristics of our sample (*n* = 35).

Characteristic	*n*	%	Mean (range)
Male	24	68.6	
Female	11	31.4	
Age (years)			64 (41–87)
Type of diabetes			
T2D	28	80	
T1D	7	20	
Duration of diabetes (years since diagnosis)			18 (<1–54)
Previous DFU			
One	14	40	
More than one	21	60	
Ethnicity			
White British	33	94.3	
Other	2	5.7	
People in household			
Living alone	11	31.4	
Two	14	40	
Three	7	20	
Four or more	3	8.6	
Employment status			
Employed	10	28.6	
Retired	16	45.7	
Carer	2	5.7	
Economically inactive	6	17.1	
Unemployed	1	2.9	
Other complications of diabetes (self-reported)			
None	9	25.7	
One	16	45.7	
Two	7	20	
Three or more	3	8.6	
Other health conditions (self-reported)			
None	2	5.7	
One	6	17.1	
Two	10	28.6	
Three or more	17	48.6	
Interviewees per recruitment site			3 (1–11)

### Theme 1: A spectrum of embodied experiences

#### ‘You go through it and you come out the other side’

Several interviewees recalled a recent (or first) DFU which had responded well to treatment, healing after a few weeks of care or a short course of antibiotics. These interviewees described these DFUs as having been unexpected, and unwelcome, but having caused only short-term discomfort and/or minor inconvenience:

Apart from changing the dressing every other day, which was no trouble at all really, and not being able to shower. . .for a couple of weeks. . . it didn’t really affect me that much. (Alex: male, 64 years-old)

Often, such interviewees appeared to have given little thought to the causes of the ulcer and to be relatively unconcerned about the possibility of developing another. Correspondingly, these interviewees expressed minimal interest in what they might do to reduce the risk of further DFUs occurring and/or escalating. Some, like Joe, appeared to assume that if they were to develop a further DFU, it would resolve similarly quickly and easily:

The old antibiotics got rid of it pretty rapidly. Now I know I can always rely on them, if I get another one. . .Hopefully I won’t! (Joe: male, 71 years-old)

#### ‘It did take over your life’

Other interviewees described more disruptive experiences, highlighting the disabling impact of an active DFU and, sometimes, severe discomfort. Some recalled difficulties walking. Others observed how they had had to give up valued activities such as driving or swimming. Instead, as Sidney recollected, life had come to revolve around day-to-day wound care and appointments at the foot clinic:

Getting up in the morning. . .you had to put dressings on, and you had to put special shoes (on), or don whatever. And. . .everything became foot-centric. . .that was the problem. It did take over your life. (Sidney: male, 68 years-old)

Typically, these interviewees described their DFUs as resolving only after some months of care and multiple courses of antibiotics. Hence, Liz related, she had started to question whether healing would ever be achieved. Several others observed how to bring about healing, surgical procedures such as revascularisation (to improve circulation) and/or minor amputations (e.g. removal of a toe) had been required. Often these interviewees appeared shaken by the experience, and grateful to have healed. Prior to enrolling in the trial, however, they had not always considered the possibility of reoccurrence and what they might do to reduce the risk of that happening. Jim, for instance, described how he had been shocked to read in the trial paperwork that:

They could come back, or they could go on to the other foot, or whatever. . .When I read that, I was thinking, Oh my goodness. I really don’t want that to happen! (Jim: male, 67 years-old)

#### ‘I don’t wanna wind up in hospital again’

A further group described how refractory and/or recurrent DFUs had curtailed and over-shadowed their lives for many years:

The last ulcer I’ve had since 2014. . .(I) must have had eight different types of infections. . .(and) an amputation of my big toe on my left foot. . .The last infection they said. . .“there’s only penicillin (left to try)”. . .Luckily, the penicillin worked. (Rick: male, 56 years-old)

These interviewees emphasised how they were acutely aware of the risks DFUs posed to their health. They highlighted how, in light of their highly protracted and/or repeated experience(s) of DFUs, their understanding of the importance of foot-care (including rapid help-seeking) had grown, along with their knowledge of what actually to do. For example, Jon, who (like many in this group) had been admitted with sepsis previously, explained how he now knew that he had to act decisively on finding signs of ulceration, and that – critically – in his locality the walk-in clinic offered the fastest route into care:

(Three years ago) I was in (hospital) and on antibiotics. . .for about a week and a half. And this time. . .within. . .a matter of hours. . .the sore arose. I could see this blister. . .I just felt this wetness on my foot. And. . .I thought, “Right, I don’t wanna wind up in hospital again. . .we’ll go down to the walk-in centre (now)!” (Jon: male, 61 years-old)

### Theme 2: Intersection with wider experiences of ill-health

#### ‘(Diabetes) doesn’t affect my life really’

Some interviewees with T2D perceived themselves as relatively unaffected by the condition, describing it as having little impact on their overall health, wellbeing and lives. Several of these interviewees described being diagnosed relatively recently, in some cases on presenting with a DFU. Others, who had been diagnosed for longer, viewed their blood glucose levels as good, sometimes questioning the legitimacy of their diagnosis. Such interviewees often used qualifiers when referring to their diagnostic status, including *‘prospective diabetic’* (William), ‘*borderline*’ (Mary) and *‘virtually not diabetic’* (Rob). The development of a DFU, with neuropathy as a precursor, did not appear to challenge such assessments. Instead, such interviewees simply suggested that their ulcer had causes other than diabetes. This appeared to inform their expectations of (DFU) reoccurrence:

Mine weren’t diabetic-related. . .it was all circulatory, (so) providing I keep my circulation OK, I should. . .be fine. (Rob: male, 57 years-old)

#### ‘Diabetes. . .affects everything. . .feet are just part of it’

Other interviewees, in contrast, described a variety of ways they were affected by diabetes. These individuals reported experiencing a range of diabetic complications, some very serious, including retinopathy, kidney problems and myopathy: *‘All the other things associated with diabetes are happening to me’*, (Alex). They identified various ways in which those complications had or could affect the development, identification and effective treatment of DFUs. A few linked the emergence of their DFU(s) to another complication and/or its management: *‘The foot ulcer. . .was a secondary by-product of being in the hospital’* (Dev, admitted following kidney failure). Others described, in detail, how their various complications required time, attention and sometimes the support of family members to manage. As a result they might, on occasion, de-prioritise or overlook foot-care. Several also noted how diabetes-related impairments to vision introduced significant practical barriers to effective, independent foot-checking. More unusually, interviewees identified other complications as having affected treatment of a DFU. Joanne, for instance, recalled how her gastrointestinal complications impeded absorption of oral antibiotics, allowing her foot infection to escalate: *‘I needed (antibiotics) . . . really fast, and my stomach just doesn’t allow that’.* Dev summed up this intersection of different complications neatly as *‘the domino effect’* – where one issue triggered or exacerbated another, accelerating overall decline.

#### ‘I’ve got loads of other. . .things wrong’

In addition, interviewees (of all ages) often reported a variety of comorbidities. These included: cardiovascular disease, respiratory illness, auto-immune disease, cancer and mental health difficulties. Several interviewees emphasised the multiplicity of health challenges they had experienced:

I’ve had a major heart attack and. . .open heart surgery. My blood pressure’s up. . .I’m riddled with osteoarthritis, and I’ve had colon cancer, burst appendix, had some of me stomach removed. (Derek: male, 67 years-old)

Whilst some interviewees downplayed the significance of these other issues, others highlighted their life-changing effects. Often, such interviewees described restrictive and unpleasant symptoms, which, they explained, individually or cumulatively impeded their ability to care for their feet (or indeed to self-manage their diabetes more generally). Several, for instance, described how limited mobility (due to age-related changes, sciatica, and/or inflammatory arthritis) made it difficult to clean, check and/or moisturise their feet:

It’s a struggle now, to get down to wash my feet. . .when I get showered, I can’t get down. . .(so) I’ll get a bowl, with probably a bit of Dettol in it, and sit at settee and wash my feet like that. . .I try to look at my feet. But I’m limited to what I can do. (Jack: male, 76 years-old)

Others detailed how mental health difficulties had disrupted their diabetes management (blood glucose control), consistency of foot-care, and/or attendance at routine podiatry appointments. Rob explained how mental health difficulties had also delayed him seeking help on developing a DFU:

I’ve had some quite bad mental health issues over the years. . .And there was something going on in my life that. . .sent me down a rabbit hole. . .So I didn’t go and get my feet done. And this thing (DFU) got worse. (Rob: male, 57 years-old)

Finally, several observed how the treatment of one condition could hinder the (clinical) management of another. Jenny, for example, explained how surgery to her foot (a minor amputation) had had to be postponed, due to being given steroids for a respiratory condition.

### Theme 3: Framing by broader life circumstances

#### Occupational/domestic demands: ‘I’m constantly working and I’m on my feet all day’

Some interviewees portrayed their occupational and/or domestic (e.g. caregiver) roles as enabling or encouraging health, highlighting how these provided a sense of purpose and connection. Several in ‘white-collar’ employment described flexible and supportive arrangements (e.g. home-working) which helped facilitate DFU-healing. Others in more physical occupations (e.g. steel worker, warehouse operative) talked of supportive colleagues, and managers who were mindful of their needs and limitations. Often, however, interviewees described work or caregiving demands which were detrimental to their general- and/or foot-health. Several highlighted how these prompted health-harming behaviours (e.g. smoking, reliance on ultra-processed/high-sugar foods) and left them short of time for foot- and self-care. Many also highlighted the long hours they routinely spent on their feet, linking this to the onset or slow healing, of a DFU. For example, Tina, a sales assistant and unpaid carer, explained how her inability to rest (and reduce loading on the affected foot) had made a marked difference to her experiences of DFUs:

(When) Covid hit last year. . .I had to self-isolate, so I had 12 weeks off. . .(and) I (had) an infected toe, on my right side, while I was off. . .but it went within five weeks. . .When I got the other infected toe . . . (that) took a lot longer to heal, ‘cause I’m constantly working and I’m on my feet all day. (Tina: female, 49 years-old)

Several also observed how specific workplace incidents had left them with vulnerabilities. Some such as Joe, a retired engineer, described how historic injuries had left them with impaired sensitivity and reduced awareness of issues with their feet:

A forklift truck ran over one of me feet. . .so I’ve probably got a circulation problem there. . .I can’t move my big toe at all. . .it’s numb on one side, completely. . .I probably would have noticed the (wound/DFU) a bit earlier, if I could’ve felt it. (Joe: male, 71 years-old)

Others meanwhile explained how historic back injuries (from lifting heavy objects or people) now impeded effective foot-care.

#### Social support: ‘It’s been very hard. . .I’ve had to rely on people’

Interviewees also highlighted how the availability or otherwise of social support had influenced their DFU experiences. Many reported receiving, and relying upon, assistance with various activities of daily living. These activities included housework, grocery shopping, dog walking and other weight-bearing tasks. A smaller group described how trusted others had identified signs of a DFU, prompted help-seeking, and/or performed essential foot-care. In some cases, this included changing interviewees’ wound dressings, reducing their need for healthcare appointments:

My sister is a trained nurse, who’s just retired. . ., so they allowed me to have her dress it. . .I didn’t have to go to hospital. My sister came round every two or three days and re-dressed it. . .for (about) a year. (Ray: male, 63 years-old)

Several such interviewees further observed how, following healing, a close family member (typically a spouse or resident adult child) had continued to help them wash, dry, exfoliate, check, and cream their feet.

Notably, however, not everyone had access to this sort of support. A substantial minority of interviewees lived alone; many of these, typically older men, also portrayed themselves as socially isolated. They attributed this situation to retirement, dispersed or fractured families, and/or bereavement. Some noted how their isolation had been compounded by the development, and restrictions of living with, a DFU. These interviewees highlighted the practical challenges of living alone with few, if any, people they could turn to for help (with daily activities or foot-care). Several noted how, although advised to keep the weight off their ulcerated foot to promote healing, it was impossible to do this diligently when there was no-one there to make a cup of tea, prepare a meal or keep the house in order:

Of course, you’re doing bits of walking, just walking around the house . . . going to the kitchen, doing little bits of housework, and so on. (Jim: male, 67 years-old)

#### Financial resources: ‘A lot of people just don’t have. . .the means’

Interviewees also highlighted diverse, sometimes considerable, expenses associated with optimal management of DFUs and preventive foot-care practices. Widely reported outlays included the purchase of higher-quality socks and/or new shoes, in order to: minimise the risk of DFU reoccurrence; avoid wearing *‘dreadful’* hospital-issue shoes; or accommodate orthopaedic insoles:

I literally have spent. . .£200, £300, on new shoes over the past year, to take into account the insoles that (County) NHS gave me. . .Now, with the new insoles, I’m going to have to do the same thing again, because they’re. . .fatter, wider. (Charles: male, 56 years-old)

In addition, several interviewees reported having paid to see a podiatrist/chiropodist, having not been offered, or having been denied access to, an ‘*overwhelmed*’ (Matthew) community podiatry service. Some also reported incurring significant transport costs (e.g. taxi fares) to attend hospital and other appointments. Harry recalled hiring a mobility scooter, and a few such as Jim, a retired teacher who developed a DFU on his left foot, described eventually investing in an automatic car to facilitate mobility and independence.

Not everyone, however, could afford these sorts of outlays. Alex explained how he had stopped seeing his chiropodist when prices increased (*‘I used to go to a chiropodist, right, but he got ever so expensive’.*) Other interviewees’ accounts suggested significant financial insecurity and/or difficulties meeting the basic costs of living (e.g. housing, heating, food). Dev explained how he had had to give up work following kidney failure, and was struggling to find a new job that would fit around his dialysis schedule. Katie, meanwhile, described how following a recent relationship breakdown she was *‘having to use a food bank’.* Replacing their footwear, seeing a podiatrist privately or buying an automatic car, were thus unrealistic options for these individuals.

#### Service access: ‘You don’t have a panic button to press until you have a problem’

Finally, interviewees’ accounts suggested substantial geographic variation in access to appropriate healthcare. Some interviewees described quick and easy access to a primary care professional, and smooth onwards referral to a diabetic foot specialist (and/or other appropriate acute service). However, others reported significant difficulties getting an appointment with a General Practitioner (GP) or practice nurse, and suggested that delays in getting seen had contributed to the severity and duration of their DFUs:

(When) I got this problem with me foot a few months ago. . .(I) got in touch with the doctor’s, “Oh, we can’t fit you in. . .” It must have been getting on for three weeks and (I) still couldn’t get an appointment, even though I said, “Look, I’m diabetic, (and) me toe’s all swollen.”. . .I was due to see my. . .chiropodist, and. . .she said, “It’s all infected!” So she took a photo, told me to go straight round to the GP’s, and (then) they sent me straight up to the hospital.” (Susan: female, 62 years-old)

Whilst Susan, above, attributed difficulties accessing primary care to the merger of two local GP practices, many others highlighted impacts of the Covid-19 pandemic. Pilot trial interviewees reported difficulties contacting staff at their practice and/or getting appointments. Main trial interviewees remarked on more enduring changes to services, including: shifts in modes of delivery (telephone appointments becoming the default); diminished support for/oversight of their diabetes management more generally; and, disruption to routine footcare services.

Notably, several interviewees observed how the development of a DFU had enhanced their access to support with foot-health (and diabetes management more generally). Those with a history of ulceration often said that this had opened up channels through which they could now access both routine (e.g. community podiatry) and acute foot-care. Critically, several observed how they could now self-refer to specialist foot-care services, via what Matthew colloquially called the ‘*panic button*’ and Mike the ‘*batphone’’*. This ability to bypass primary or community care, they suggested, shortened the pathway to specialist care significantly.

## Discussion

Previous qualitative work on experiences of DFUs has emphasised their disruptive and extended impact and attended primarily to the role, in their development and management, of intrinsic, individual factors, for example, knowledge, physical capabilities and personal choices (see, inter alia, Barg et al., 2017; Coffey et al., 2019; Costa et al., 2024; Costa and Camargo-Plazas, 2023; Crocker et al., 2021; Meriç et al., 2019; Simonsen et al., 2024; Van Netten et al., 2019). That work is valuable and has provided a useful foundation for our own. However, through close scrutiny of a large body of data, using a sociologically-informed contextual lens, we have been able to develop a more comprehensive and nuanced understanding of the diversity of lived experiences of DFUs. Pivotally, this understanding recognises and spotlights the additional and powerful influence of extrinsic, contextual factors. These factors include the social, occupational, material and – in large part geographically determined – health service-related characteristics of individuals’ lives.

In reporting our findings, we began by identifying *a spectrum of embodied experience*, which varied both between individuals and over time. This ran from short-lived episodes of ulceration, described by relevant interviewees as having minimal impact and significance, through to extended and sometimes recurrent, periods of ulceration which dominated people’s lives. The relatively sanguine accounts provided by some of our interviewees have featured only rarely in prior literature (see Aagaard et al., 2024), which has instead typically emphasised more dramatic experiences (see, inter alia, Barg et al., 2017; Beattie et al., 2014; Costa et al., 2024; Costa and Camargo-Plazas, 2023; Marchand et al., 2012; Searle et al., 2008). We hold that less impactful experiences also deserve attention, as, notwithstanding a relatively mild first presentation, affected individuals remain at significant risk of re-ulceration (Boulton, 2008). Moreover, interviewees’ accounts suggest that, in the absence of appropriate information and support, future ulceration risk might not be managed optimally. Indeed, instead of prompting enhanced self-care, interviewees’ accounts suggested that an easily-resolved DFU could encourage complacency. Markedly different perspectives were shared by interviewees further along the spectrum. Those accounts revealed how views on the significance and potential impact of DFUs were informed by, and evolved alongside, shifts in embodied experience. Notably, interviewees who had had DFUs which were sufficiently serious as to require invasive procedures and/or hospitalisation described ‘tipping points’ in their perceptions of, and responses to, changes in the condition of their feet. These shifts in perspective echo what O’Connor et al. (1997) termed a ‘conversion experience’. They also evoke Lawson and Flocke (2009)’s concept of ‘teachable moments’, or situations that invite behaviour change – and which might be capitalised upon by healthcare professionals seeking to bring such change about.

Next, we attended to the *intersection of (DFU) experiences with wider experiences of ill-health*. Whilst some of our interviewees dismissed their diabetes diagnosis as being of little significance, others detailed numerous diabetes-related complications, each requiring time and attention. Many also described other health conditions, sometimes very serious, with their own management demands. These could over-shadow, and lead individuals to de-prioritise, recommended foot-care practices. Such accounts bring to mind May et al.’s (2014) conceptualisation of how the accumulating work associated with chronic illness(es) may lead to overwhelm, with this precipitating poorer health outcomes (and increased service use). We note that prior research on experiences of DFUs has been narrower (more condition-specific) in its focus (see, inter alia, Barg et al., 2017; Beattie et al., 2014; Costa et al., 2024; Costa & Camargo-Plazas, 2023; Meriç et al., 2019). Our work, in dialogue with that of May et al. (2014), demonstrates the value of exploring people’s wider health and illness experiences, in order to understand fully the challenges they may encounter with regard to managing and preventing reoccurrence of DFUs.

Finally, we explored the *framing of DFU experiences by broader life circumstances*, and, in particular how the conditions of people’s lives affected their capacity to react constructively to, and prevent, ulceration. We found that individuals’ occupational and domestic roles, access to social support, financial circumstances and the character of their local healthcare services, shaped their DFU experiences and outcomes in dramatically different ways. It is here that our work differs most from, and adds most to, the prior literature – hence the prominence this theme has in our reporting. Earlier literature, as noted above, has attended almost exclusively to intrinsic, individual factors. Drawing upon the traditions of medical sociology, our work, like that of Bury (1982, 1991) and Charmaz (1983), has illuminated the diverse ways is which social and material circumstances can affect people’s illness experiences. In so doing, it aligns with Lawton’s (2003) and Mol’s (2009) calls for closer attention to be paid to the more ‘mundane’ (physical, social and material) facets of illness experiences, and the grounding of self-care decisions in the ‘lifeworld.’

The World Health Organization has similarly argued for greater recognition of the impact on health of people’s socio-economic circumstances, including their access to appropriate healthcare, (CSDH, 2008). Research in other clinical areas has shown the profound impact of service organisation and delivery on individuals’ health behaviours and outcomes, with Hart (2001)’s concept of ‘system-induced setbacks’ being particularly salient. Several interviewees in our study reported great difficulty accessing primary care, and held this accountable for the escalation of their foot problems and subsequently protracted healing. Such perspectives find support in both the diabetes epidemiology literature (McDermott et al., 2023) and a recent audit of diabetes footcare in England and Wales (NHS England, 2024). That audit identified time to ‘first expert assessment’ as pivotal to patient outcomes, and concluded that outcomes were significantly better where people could self-refer for expert assessment, for example, using what one of our interviewees called ‘a panic button’.

### Implications for practice

Our findings, whilst sociologically-informed, have clear implications for practice. Firstly, depending on the character of their embodied experience(s), people are likely to perceive DFU risk differently – and to vary in their readiness to change their behaviour to manage that. Hence, individuals with a relatively mild (rapidly resolving) first incident might be expected have quite different information and support needs to those who have had more disturbing and impactful DFU experiences. Secondly, foot-care professionals need to be sensitive to the challenges to foot-care other complications and/or comorbidities present (e.g. impaired vision, restricted movement) as well as the competing care burdens which some individuals have to juggle. People may need considerable, tailored support and encouragement to surmount and/or reconcile such demands. Thirdly, as the social and material circumstances of people’s lives can affect their capacity to manage and prevent DFUs in a wide variety of ways, a ‘one-size-fits-all’ approach to enhancing foot-care behaviours is unlikely to be effective. Interventions (such as REDUCE) which are tailored to individuals’ circumstances *may* have a better chance of success. However, these too have limited power to change the fundamental character of people’s lives (e.g. living alone, in poverty) or to address important structural issues, in particular the availability of local health/foot-care services.

### Strengths and limitations

Before closing, we consider how our methodological choices may have shaped our findings. Our work differed from many precursor studies in its scale (the majority of the studies synthesised by Coffey et al. (2019) involved ⩽15 participants, drawn from 1 to 2 sites) and from all in its context-sensitive approach. Arguably, our comparatively large sample, purposively constructed to include interviewees recruited from different sites, of different ages and genders, and with different clinical histories, was pivotal to uncovering the diversity in experience we report. Nevertheless, some features of our sample may have precluded uncovering the full range of experience. Firstly, all participants had recently-healed ulcers at the time of recruitment to the study; their perspectives on the past may have been influenced by experiences in/of the present. Secondly, the sample’s limited ethnic diversity, whilst reflective of the associated patient population (Abbott et al., 2005; Pham et al., 2019) leaves questions unanswered regarding cultural aspects of context. Wider work, we should note, has documented how culturally-informed footwear practices may increase the risk of foot injury, (Pérez-Belloso et al., 2022) whilst clothing choices may discourage physical activity (Lawton et al., 2006b). Also noteworthy is that our interviews, although conducted remotely, were lengthy, wide-ranging and revealing. This was likely facilitated by both interviewers having many years (20-plus each) of qualitative research experience, including interviewing by telephone or video-call. In addition, it may be salient that early interviews were conducted when the UK was in ‘lockdown’, and interviewees had the time and inclination to talk to someone who appeared, and was, genuinely interested in their lives.

## Conclusions

Experiences of DFUs are far more nuanced and contextually-mediated than previously reported. This has important practical implications for the provision of sensitive and effective support and clinical care. Identifying complexity in illness experiences may require larger, and more diverse samples and data-sets than have become the norm in qualitative health research. In addition, if we are to understand the wider contexts within which illness experiences play out, it may be necessary to adopt a more expansive disciplinary lens.

## Supplemental Material

sj-docx-1-hea-10.1177_13634593251387548 – Supplemental material for Experiences of living with, managing, and preventing reoccurrence of Diabetic Foot Ulcers: Restoring context and complexity to health and illness research Supplemental material, sj-docx-1-hea-10.1177_13634593251387548 for Experiences of living with, managing, and preventing reoccurrence of Diabetic Foot Ulcers: Restoring context and complexity to health and illness research by Ruth I. Hart, David Rankin, Fran Game, Kavita Vedhara and Julia Lawton in Health

## References

[bibr1-13634593251387548] Aagaard TV Skou ST Brorson S , et al (2024) Patients’ behaviour after referral to a wound care clinic for diabetic foot ulcer care: A grounded theory study. Journal of Wound Care 33(6): 432–440. 38843012 10.12968/jowc.2022.0265

[bibr2-13634593251387548] Abbott CA Garrow AP Carrington AL , et al (2005) Foot ulcer risk is lower in South-Asian and African-Caribbean compared with European diabetic patients in the UK: The North-West Diabetes Foot Care Study. Diabetes Care 28(8): 1869–1875. 16043725 10.2337/diacare.28.8.1869

[bibr3-13634593251387548] Armstrong DG Boulton AJM Bus SA (2017) Diabetic foot ulcers and their recurrence. New England Journal of Medicine 376(24): 2367–2375. 28614678 10.1056/NEJMra1615439

[bibr4-13634593251387548] Barg FK Cronholm PF Easley EE , et al (2017) A qualitative study of the experience of lower extremity wounds and amputations among people with diabetes in Philadelphia. Wound Repair and Regeneration 25(5): 864–870. 29220878 10.1111/wrr.12593

[bibr5-13634593251387548] Bate P (2014) Context is everything. In: Bamber J (ed.) The Health Foundation. Perspectives on Context: A Selection of Essays Considering the Role of Context in Successful Quality Improvement. The Health Foundation, pp. 1–29.

[bibr6-13634593251387548] Beattie AM Campbell R Vedhara K (2014) ‘What ever I do it’s a lost cause.’ the emotional and behavioural experiences of individuals who are ulcer free living with the threat of developing further diabetic foot ulcers: A qualitative interview study. Health Expectations 17: 429–439. 22429399 10.1111/j.1369-7625.2012.00768.x PMC5060729

[bibr7-13634593251387548] Boulton AJM (2008) The diabetic foot: Grand overview, epidemiology and pathogenesis. Diabetes/Metabolism Research and Reviews 24(S1): S3–S6. 10.1002/dmrr.833 18442166

[bibr8-13634593251387548] Braun V Clarke V (2006) Using thematic analysis in psychology. Qualitative Research in Psychology 3: 77–101.

[bibr9-13634593251387548] Bury M (1982) Chronic illness as biographical disruption. Sociology of Health & Illness 4(2): 167–182. 10260456 10.1111/1467-9566.ep11339939

[bibr10-13634593251387548] Bury M (1991) The sociology of chronic illness: A review of research and prospects. Sociology of Health & Illness 13(4): 451–468.

[bibr11-13634593251387548] Charmaz K (1983) Loss of self: A fundamental form of suffering in the chronically ill. Sociology of Health & Illness 5(2): 168–195. 10261981 10.1111/1467-9566.ep10491512

[bibr12-13634593251387548] Coffey L Mahon C Gallagher P (2019) Perceptions and experiences of diabetic foot ulceration and foot care in people with diabetes: A qualitative meta-synthesis. International Wound Journal 16: 183–210. 30393976 10.1111/iwj.13010 PMC7949356

[bibr13-13634593251387548] Cornwell J (1984) Hard-Earned Lives: Accounts of Health and Illness From East London. Tavistock Publications.

[bibr14-13634593251387548] Costa D Gallelli G Scalise E , et al (2024) Socio-cultural aspects of diabetic foot: An Ethnographic study and an integrated model proposal. Societies 14: 240.

[bibr15-13634593251387548] Costa IG Camargo-Plazas P (2023) The impact of diabetic foot ulcer on individuals’ lives and daily routine: A qualitative study informed by social constructivism and symbolic interactionism frameworks. Journal of Wound Ostomy and Continence Nursing 50(1): 73–77. 10.1097/WON.0000000000000941 36640167

[bibr16-13634593251387548] Costa IG Tregunno D Camargo-Plazas P (2020) I cannot afford off-loading boots: Perceptions of socioeconomic factors influencing engagement in self-management of diabetic foot ulcer. Advances in Nursing Science 43(4): 322–337. 32956088 10.1097/ANS.0000000000000328

[bibr17-13634593251387548] Crocker RM Palmer KNB Marrero DG , et al (2021) Patient perspectives on the physical, psycho-social, and financial impacts of diabetic foot ulceration and amputation. Journal of Diabetes and Its Complications 35(8): 107960. 34059410 10.1016/j.jdiacomp.2021.107960 PMC8316286

[bibr18-13634593251387548] CSDH (2008) Closing the gap in a generation: health equity through action on the social determinants of health. Final Report of the Commission on Social Determinants of Health. World Health Organization.

[bibr19-13634593251387548] Diabetes UK (2024) How to look after your feet. Available at: https://www.diabetes.org.uk/about-diabetes/looking-after-diabetes/complications/feet/taking-care-of-your-feet (accessed 27 October 2025).

[bibr20-13634593251387548] Emslie C Hunt K Watt G (2001) Invisible women? The importance of gender in lay beliefs about heart problems. Sociology of Health & Illness 23(2): 203–233.

[bibr21-13634593251387548] Hart E (2001) System induced setbacks in stroke recovery. Sociology of Health & Illness 23(1): 101–123.

[bibr22-13634593251387548] Hjelm K Nyberg P Apelqvist J (2002) Gender influences beliefs about health and illness in diabetic subjects with severe foot lesions. Journal of Advanced Nursing 40(6): 673–684. 12473048 10.1046/j.1365-2648.2002.02427.x

[bibr23-13634593251387548] Kerr M Barron E Chadwick P , et al (2019) The cost of diabetic foot ulcers and amputations to the National Health Service in England. Diabetic Medicine 36(8): 995–1002. 31004370 10.1111/dme.13973

[bibr24-13634593251387548] Lawson PJ Flocke SA (2009) Teachable moments for health behavior change: A concept analysis. Patient Education and Counseling 76(1): 25–30. 19110395 10.1016/j.pec.2008.11.002 PMC2733160

[bibr25-13634593251387548] Lawton J (2002) Colonising the future: temporal perceptions and health-relevant behaviours across the adult lifecourse. Sociology of Health & Illness 24(6): 714–733.

[bibr26-13634593251387548] Lawton J (2003) Lay experiences of health and illness: Past research and future agendas. Sociology of Health & Illness 25(3): 23–40. 14498928 10.1111/1467-9566.00338

[bibr27-13634593251387548] Lawton J Ahmad N Hanna L , et al (2006a) Diabetes service provision: A qualitative study of the experiences and views of Pakistani and Indian patients with type 2 diabetes. Diabetic Medicine 23: 1003–1007. 16922707 10.1111/j.1464-5491.2006.01922.x

[bibr28-13634593251387548] Lawton J Ahmad N Hanna L , et al (2006b) ‘I can’t do any serious exercise’: Barriers to physical activity amongst people of Pakistani and Indian origin with Type 2 diabetes. Health Education Research 21(1): 43–54. 15955792 10.1093/her/cyh042

[bibr29-13634593251387548] Lawton J Ahmad N Hanna L , et al (2008b) “We should change ourselves, but we can’t”: Accounts of food and eating practices amongst British Pakistanis and Indians with type 2 diabetes. Ethnicity and Health 13: 305–319. 18701991 10.1080/13557850701882910

[bibr30-13634593251387548] Lawton J Peel E Parry O , et al (2005) Lay perceptions of type 2 diabetes in Scotland: bringing health services back in. Social Science & Medicine 60: 1423–1435. 15652676 10.1016/j.socscimed.2004.08.013

[bibr31-13634593251387548] Lawton J Peel E Parry O , et al (2008a) Shifting accountability: A longitudinal qualitative study of diabetes causation accounts. Social Science & Medicine 67: 47–56. 18440113 10.1016/j.socscimed.2008.03.028

[bibr32-13634593251387548] Littman AJ Young J Moldestad M , et al (2021) How patients interpret early signs of foot problems and reasons for delays in care: Findings from interviews with patients who have undergone toe amputations. PLoS One 16(3): e0248310. 10.1371/journal.pone.0248310 PMC7946282 33690723

[bibr33-13634593251387548] Marchand C Ciangura C Griffe V , et al (2012) Barriers to preventive and curative foot care behaviors in person with diabetes. Suggestions for therapeutic patient education. Education Thérapeutique du Patient - Therapeutic Patient Education 4: S135–S142.

[bibr34-13634593251387548] Maxwell JA (2012) A Realist Approach for Qualitative Research. Sage.

[bibr35-13634593251387548] May CR Eton DT Boehmer K , et al (2014) Rethinking the patient: Using burden of treatment theory to understand the changing dynamics of illness. BMC Health Services Research 14: 281. 24969758 10.1186/1472-6963-14-281 PMC4080515

[bibr36-13634593251387548] McDermott K Fang M Boulton AJM , et al (2023) Etiology, epidemiology, and disparities in the burden of diabetic foot ulcers. Diabetes Care 46(1): 209–221. 36548709 10.2337/dci22-0043 PMC9797649

[bibr37-13634593251387548] Meriç M Ergün G Meriç C , et al (2019) It is not diabetic foot: It is my foot. Journal of Wound Care 28(1): 30–37. 30625047 10.12968/jowc.2019.28.1.30

[bibr38-13634593251387548] Mol A (2009) Living with diabetes: Care beyond choice and control. Lancet 373: 1756–1757. 19480076 10.1016/s0140-6736(09)60971-5

[bibr39-13634593251387548] NHS England (2024) National Diabetes Foot Care Audit Report 2018 to 2023. Available at: https://digital.nhs.uk/data-and-information/publications/statistical/national-diabetes-footcare-audit/2018-2023 (accessed 4 September 2024).

[bibr40-13634593251387548] O’Connor PJ Crabtree BF Yanoshik MK . (1997) Differences between diabetic patients who do and do not respond to a diabetes care intervention: A qualitative analysis. Family Medicine 29: 424–428. 9193915

[bibr41-13634593251387548] Pawson R Tilley N (1997) Realistic Evaluation. Sage Publications Ltd.

[bibr42-13634593251387548] Pérez-Belloso AJ Montaño-Jiménez P Algaba-Del-Castillo J , et al (2022) Impact of foot health behavior among ethnic minority populations: A cross-sectional population-based study. Public Health Nursing 39: 736–743. 34981857 10.1111/phn.13043

[bibr43-13634593251387548] Pham TM Carpenter JR Morris TP , et al (2019) Ethnic differences in the prevalence of type 2 diabetes diagnoses in the UK: Cross-sectional analysis of the Health Improvement Network Primary Care Database. Clinical Epidemiology 11: 1081–1088. 32021464 10.2147/CLEP.S227621 PMC6948201

[bibr44-13634593251387548] Rodrigues BT Vangaveti VN Urkude R , et al (2022) Prevalence and risk factors of lower limb amputations in patients with diabetic foot ulcers: A systematic review and meta-analysis. Diabetes & Metabolic Syndrome Clinical Research & Reviews 16: 102397. 10.1016/j.dsx.2022.102397 35085918

[bibr45-13634593251387548] Rogers L De Brún A McAuliffe E (2020) Defining and assessing context in healthcare implementation studies: A systematic review. BMC Health Services Research 20: 591. 32600396 10.1186/s12913-020-05212-7 PMC7322847

[bibr46-13634593251387548] Searle A Gale L Campbell R , et al (2008) Reducing the burden of chronic wounds: Prevention and management of the diabetic foot in the context of clinical guidelines. Journal of Health Services Research & Policy 13(Suppl. 3): 82–91. 18806197 10.1258/jhsrp.2008.008011

[bibr47-13634593251387548] Simonsen MB Christiansen SL Pedersen MK , et al (2024) Health literacy and cognitive function in people with diabetic foot ulcer with focus on knowledge, attitude, and practice in relation to foot self-care. Sage Open Medicine 12: 1–12. 10.1177/20503121241258841 PMC11159546 38855003

[bibr48-13634593251387548] Strauss A Corbin J (1990) Basics of Qualitative Research: Grounded Theory Procedures and Techniques. Sage Publications Inc.

[bibr49-13634593251387548] Tong A Sainsbury P Craig J. (2007) Consolidated criteria for reporting qualitative research (COREQ): a 32-item checklist for interviews and focus groups. International Journal for Quality in Health Care 19(6): 349–357. 17872937 10.1093/intqhc/mzm042

[bibr50-13634593251387548] Van Netten JJ Seng L Lazzarini PA , et al (2019) Reasons for (non-)adherence to self-care in people with a diabetic foot ulcer. Wound Repair and Regeneration 27(5): 530–539. 31107578 10.1111/wrr.12728

[bibr51-13634593251387548] Walsh JW Hoffstad OJ Sullivan MO , et al (2016) Association of diabetic foot ulcer and death in a population-based cohort from the United Kingdom. Diabetic Medicine 33: 1493–1498. 26666583 10.1111/dme.13054

[bibr52-13634593251387548] Whicher CA O’Neill S Holt RIG . (2020) Diabetes in the UK: 2019. Diabetic Medicine 37(2): 242–247. 31901175 10.1111/dme.14225

[bibr53-13634593251387548] Williams SJ (1999) Is anybody there? Critical realism, chronic illness and the disability debate. Sociology of Health & Illness 21(6): 797–819.

