# Supplementary material for: Experiences of living with, managing, and preventing reoccurrence of Diabetic Foot Ulcers: Restoring context and complexity to health and illness research
Source: Health (London). 2025 Nov 8;30(4):558–77. doi: 10.1177/13634593251387548 (PMC13272836; doi:10.1177/13634593251387548)
Supplement: sj-docx-1-hea-10.1177_13634593251387548 – Supplemental material for Experiences of living with, managing, and preventing reoccurrence of Diabetic Foot Ulcers: Restoring context and complexity to health and illness research [file sj-docx-1-hea-10.1177_13634593251387548.docx]

**COREQ^[[1]](#footnote-1)^ CHECKLIST (SUPPLEMENTARY FILE 1)**

| **ITEM** | | **ILLUSTRATIVE CONTENT** | **IF/WHERE AVAILABLE** |  |
| --- | --- | --- | --- | --- |
| **Domain 1: Research team and reflexivity** | | | |  |
| ***Personal Characteristics*** | | | |  |
| 1. Interviewer(s) / facilitator(s) | | Details of who conducted the interviews or focus groups | p.4* |  |
| 2. Credentials | | Details of researchers’ qualifications (e.g., PhD, MD) | Not detailed in the manuscript, but this information is widely available (see, for example, [Ruth Hart \| The University of Edinburgh](https://edwebprofiles.ed.ac.uk/profile/ruth-hart)). |  |
| 3. Occupation | | Details of researchers’ occupations | p.4 and biographical note. |  |
| 4. Gender | | Details of researchers’ gender | p.4 |  |
| 5. Experience and training | | Details of researchers’ experience and training | p.4, p.14, and biographical note. |  |
| 6. Relationship established | | Details of prior relationship between researcher and participant(s), if any | Not explicitly stated in the manuscript, but the lack of any prior relationship can be deduced from information provided on pp.3-4. |  |
| 7. Participant knowledge of the interviewer | | Details of participants' knowledge of the researchers (e.g., background, role and purpose) | Not discussed in the manuscript, due to word constraints.  Information about the research team was provided in the PIS, whilst information about the relevant research interviewer was provided verbally, ahead of interview. |  |
| 8. Interviewer characteristics | | Other information about the researchers | p.4, p.14, and biographical note. |  |
| **Domain 2: study design** | | |  |  |
| ***Theoretical framework*** | | |  |  |
| 9. Methodological orientation and Theory | Outline of theoretical underpinnings: the ontology, epistemology, and methodology informing the work | pp.3-5 |  |  |
| ***Participant selection*** | | |  |  |
| 10. Sampling | Information on the process of selecting participants | p.3 |  |  |
| 11. Method of approach | Information on how participants were recruited | pp.3-4 |  |  |
| 12. Sample size | Information on the number of participants | p.5 |  |  |
| 13. Non-participation | Information on those choosing not to participate | Not detailed in the manuscript, due to word constraints.  Information on those choosing not to participate in the trial, intervention, and/or nested qualitative study, is available from the corresponding author on reasonable request. |  |  |
| 14. Setting of data collection | Information on the site and medium of data collection | p.4 and p.14 |  |  |
| 15. Presence of non-participants | Information on who – if anyone else – was present | Not discussed in the manuscript, due to word constraints.  This was not always known with complete certainty, as interviews were conducted remotely. |  |  |
| 16. Description of sample | Characterization of the sample (e.g., demographic and clinical features) | pp.5-6 |  |  |
| ***Data collection*** | | |  | No |
| 17. Interview guide | Description of its development and content | pp.4-5, including Box 1. |  |  |
| 18. Repeat interviews | Details, where relevant | p.3 (we note only that the work was longitudinal – data from subsequent interviews did not inform this manuscript).  For information, where possible, we undertook two further interviews with participants (after they had completed or disengaged from the trialled intervention). |  |  |
| 19. Audio/visual recording | Indication of how data was captured and whether audio or video recordings were made | p.4 |  |  |
| 20. Field notes | Information on whether field notes were prepared and used | Not discussed in the manuscript, due to word constraints.  For information, notes were made and referred to subsequently, e.g., when checking/correcting transcripts. |  |  |
| 21. Duration | Information on how long interviews lasted (typically and/or range) | p.4 |  |  |
| 22. Data saturation | Discussion of data saturation | p.3 (though this term is not used). |  |  |
| 23. Transcripts returned | Indication of whether transcripts were returned to participants for comment and/or correction (and if so, what was done subsequently) | Not discussed in the manuscript, due to word constraints.  They were not returned for comment, as this was judged to add to the burden of participation. |  |  |
| **Domain 3: analysis and findings** | | |  |  |
| ***Data analysis*** | | |  |  |
| 24. Number of data coders | Details of number of people involved in analysis (and who) | p.5 |  |  |
| 25. Description of the coding tree | Discussion and/or presentation of the process of developing codes and themes | p.5 |  |  |
| 26. Derivation of themes | Indication of overarching approach to identifying themes: *a priori* or from the data | p.5 |  |  |
| 27. Software | Details of whether and what software was used to manage the data | Not discussed in the manuscript, due to word constraints.  For information, standard MS Office software used. |  |  |
| 28. Participant checking | Information on whether and how participants’ feedback on findings was sought and used. | Not discussed in the manuscript, due to word constraints.  Feedback was not sought, as this was judged to add to the burden of participation. |  |  |
| ***Reporting*** | | |  |  |
| 29. Quotations presented | Presentation of appropriate illustrative quotes with indication of their source (i.e., with participant identifier) | pp.6-11 |  |  |
| 30. Data and findings consistent | Consistency in presentation of data and findings | pp.6-11 |  |  |
| 31. Clarity of major themes | Clear presentation of principal themes | pp.6-11 |  |  |
| 32. Clarity of minor themes | Findings include consideration of minor themes (and diversity within data) | pp.6-11  The importance of capturing, attending to, and reporting diversity is one of the manuscript’s key messages. |  |  |

* All page numbers relate to the final Word document submitted to the journal for publication (i.e., the accepted version of the authors’ manuscript).

1. Tong A, Sainsbury P, Craig J. Consolidated criteria for reporting qualitative research (COREQ): a 32-item checklist for interviews and focus groups. International Journal for Quality in Health Care. 2007. Volume 19, Number 6: pp. 349 – 357 [↑](#footnote-ref-1)
